# Metal–Organic Frameworks Against Tumor Metastasis: Therapeutic Targets, Strategies, and Challenges for Clinical Translation

**DOI:** 10.1002/cam4.71349

**Published:** 2025-11-23

**Authors:** Jiahui Wang, Xiuxiu Qiu, Ruoyu Wang, Beibei Hong, Hegen Li, Zhanxia Zhang

**Affiliations:** ^1^ Cancer Institute Longhua Hospital, Shanghai University of Traditional Chinese Medicine Shanghai China; ^2^ Department of Oncology Longhua Hospital, Shanghai University of Traditional Chinese Medicine Shanghai China

**Keywords:** drug delivery systems, metal–organic frameworks, neoplasm metastasis, targeted therapy, tumor microenvironment

## Abstract

Tumor invasion and metastasis are the primary causes of cancer‐related mortality and have a profound impact on patient prognosis. This review comprehensively examines the application of Metal‐Organic Frameworks (MOFs) in anti‐metastatic therapy and establishes a theoretical foundation for their clinical translation. A systematic literature analysis clarifies the structural and therapeutic profile of MOFs. These materials show significant promise for anti‐metastatic therapy, leveraged by their high loading capacity, tunable responsiveness, and modifiable surface properties. This review discusses MOF‐based multi‐targeted strategies to combat metastasis, including tumor microenvironment modulation, targeting of key metastatic cells, and multimodal synergistic therapies. These approaches have demonstrated success in applications such as drug delivery, photodynamic therapy, and immune activation. The discussion further addresses critical challenges in biosafety and targeting efficiency for clinical translation, while recognizing how MOFs' structural versatility sustains their therapeutic development. This reviewaims to provide a theoretical foundation and practical framework for the innovative design and clinical translation of MOFs in anti‐metastatic therapy.

## 1 Introduction

Tumor invasion and metastasis are the primary causes of cancer‐related mortality, particularly in advanced stages, where metastatic spread significantly reduces overall survival and quality of life for patients [1]. During the metastatic process, tumor cells undergo a series of complex and dynamic biological transformations, including reduced cell–cell adhesion, cytoskeletal remodeling, integrin signaling reprogramming, upregulation of protease expression, as well as epithelial‐to‐mesenchymal transition (EMT) and enhanced resistance to apoptosis [2]. These changes collectively enable tumor cells to breach local tissue barriers, enter the circulatory system, and establish heterogeneous metastatic foci in distant organs [3].

Tumor metastasis is a complex, multi‐step process that involves numerous molecular mechanisms, varying across different types of tumors [4, 5, 6]. These complexities underscore the urgent need for innovative therapeutic strategies that can precisely target key steps in the metastatic cascade. Conventional treatments, such as surgery, radiotherapy, and chemotherapy, are effective for localized tumors but show limited success against metastasis due to systemic toxicity and drug resistance [7, 8]. These limitations highlight the urgent need for innovative treatments that can precisely target the complex metastatic process.

Metal Organic Frameworks (MOFs), as a class of highly ordered and structurally programmable porous materials, have demonstrated tremendous potential in the field of biomedicine in recent years [9]. MOFs possess tunable pore sizes, excellent surface areas, and enhanced drug loading and release capacities [10]. Additionally, they can be easily functionalized, allowing for targeted delivery and precise intervention at different stages of metastasis. Unlike other nanomaterials, MOFs support multiple therapeutic modalities, including chemotherapy, photothermal therapy (PTT), photodynamic therapy (PDT), and immunotherapy, enabling synergistic treatment strategies that enhance antitumor effects [11].

Although MOFs have achieved preliminary success in the treatment of primary tumors, their application in the intervention of tumor invasion and metastatic progression remains in the early stages. In particular, effective and selective targeting of the metastatic cascade, which spans local invasion, hematogenous dissemination, and distant colonization, remains a significant translational challenge. Key limitations include regulating MOFs' biodegradability, optimizing circulation stability, and enhancing targeting specificity in the context of metastatic microenvironments [12, 13, 14].

In recent years, several studies have comprehensively discussed the biomedical applications of MOFs, particularly in areas such as drug delivery, diagnostic imaging, and combined cancer therapies. Notably, recent works have provided valuable insights into the design of nanoscale MOFs and their potential in oncology, including therapeutic strategies and translational considerations [15, 16]. However, most of these studies primarily focus on MOF‐based interventions in primary tumors, with relatively limited discussion on their role in addressing metastatic disease.

In contrast, this review specifically focuses on the application of MOFs in the context of tumor invasion and metastasis, aiming to bridge this critical gap. We systematically summarize recent advances on MOFs in the treatment of tumor invasion and metastasis, discussing in detail their mechanisms of action and intervention strategies at different stages of metastasis. Additionally, we highlight the key opportunities and challenges for optimizing the design and ensuring the clinical translation of MOFs. By offering this metastasis‐centered perspective, we aim to provide a complementary and focused overview that extends existing discussions in the field and can guide future research and development in this emerging area.

## 2 Basic Characteristics of MOFs and Their Role in Tumor Invasion and Metastasis Therapy

### 2.1 Structural Features and Their Role in Antimetastatic Activity

**FIGURE 1:**
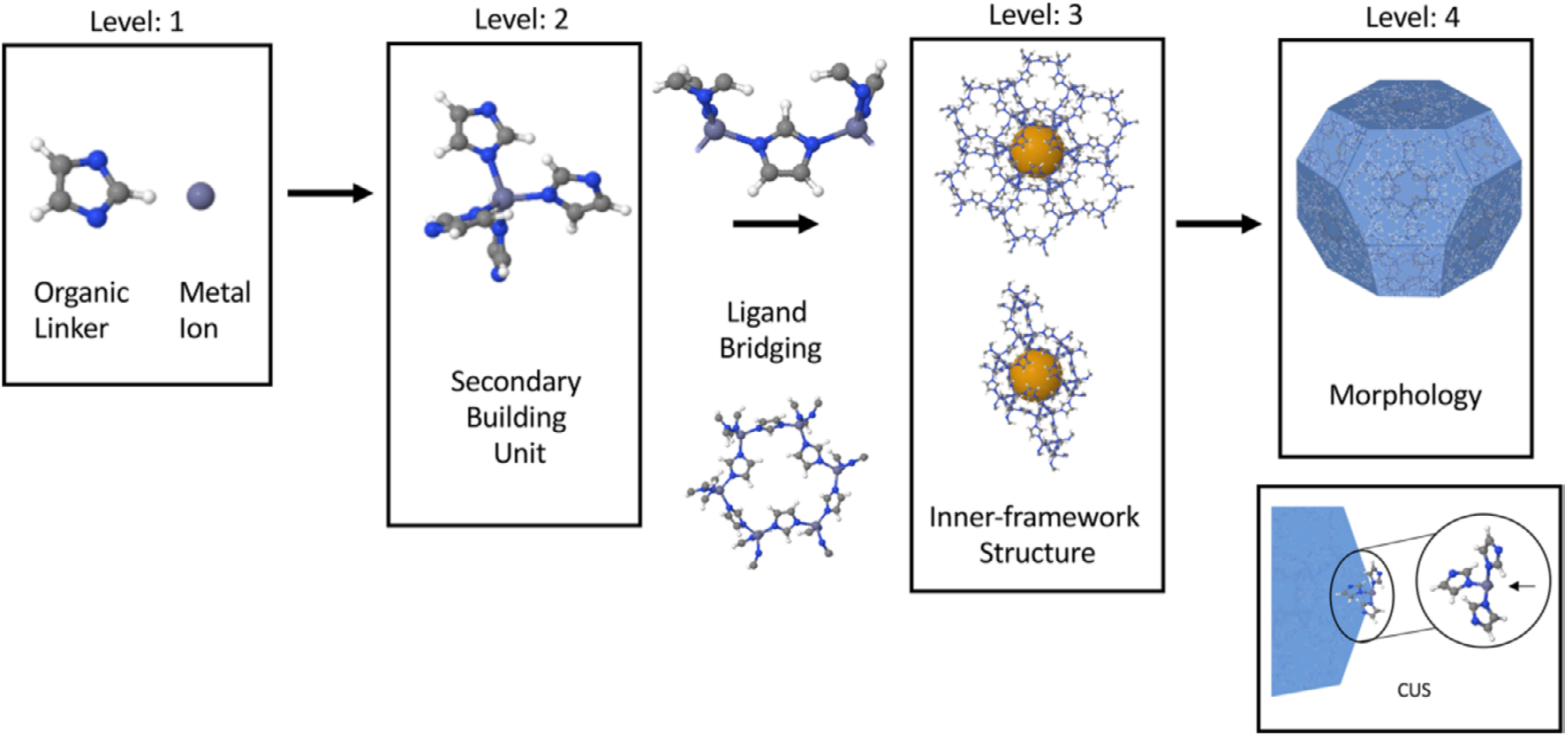
Illustration of the structural hierarchy and composition in MOFs: (1) Primary components: metal nodes and organic linkers; (2) Development of secondary building units (SBUs) and presence of coordinatively unsaturated sites (CUS); (3) Formation of the Internal Framework Architecture; (4) Resultant Particle Morphology. Molecular models are adapted with permission from ChemTube3D (https://www.ChemTube3D.com). Reprinted (adapted) with permission from [19]. Copyright © 2021, American Chemical Society.

MOFs are crystalline porous materials formed by the coordination of metal ions/clusters with organic ligands, featuring highly ordered structures and tunable porosity [17, 18, 19] (Figure 1). With their exceptional surface area, diverse pore size design, and precise compositional tunability, MOFs are extensively used in adsorption, separation, catalysis, magnetic devices, chemical sensing, and biomedicine [20, 21, 22].

MOFs exhibit a wide variety and can be classified according to various criteria based on their structural characteristics and constituent elements [23]. From the perspective of metal nodes, common metal ions include transition metals such as iron (Fe), zinc (Zn), copper (Cu), manganese (Mn), as well as some main group metals like aluminum (Al). MOFs constructed with different metal nodes exhibit distinct physicochemical properties and potential applications [24]. For example, MOFs with Zn as the metal node, such as ZIF‐8, exhibit good chemical stability and biocompatibility, and have been extensively studied for their applications in drug delivery and biological imaging [25]. Iron‐based MOFs (Fe‐MOFs) exhibit unique application potential in fields such as catalysis [26] and biomedicine [27, 28]. Owing to the unique redox characteristics of iron ions. Especially in tumor therapy, the redox activity of Fe‐MOFs holds promise for providing new mechanisms for treatment [29].

In the treatment of antitumor invasion and metastasis, the structural characteristics of MOFs provide them with unique advantages. First, their ultra‐high porosity and surface area enable MOFs to efficiently load small‐molecule drugs or macromolecular therapeutics, significantly enhancing drug delivery and local concentration, thereby inhibiting the invasive phenotype of tumor cells [30]. Additionally, the programmable design of pore size and surface chemistry enables MOFs to precisely recognize and bind tumor‐associated biomarkers, thereby enhancing the selectivity and specificity of treatment [12]. Furthermore, the excellent structural stability of MOFs ensures their functionality during in vivo transport and targeting. Certain types of MOFs, such as Zn‐MOFs, undergo controllable degradation in the acidic lysosomal environment, ensuring timely drug release while effectively reducing long‐term toxicity risks [31]. This series of structure–function relationships provides a solid foundation for the application of MOFs in intervening in tumor metastasis.

### 2.2 Application of MOFs in Antimetastatic Cancer Therapy

In recent years, the multimodal applications of MOFs in primary tumor treatment have provided insights for their expansion in metastasis prevention and control [32]. As a drug delivery platform, MOFs can achieve efficient and targeted drug release, significantly enhancing the inhibitory effect of anticancer drugs on primary tumors while reducing nonspecific toxicity to normal tissue [33]. By effectively controlling the progression of primary tumors, MOFs indirectly reduce the risk of tumor cells entering the bloodstream and colonizing distant organs.

MOFs demonstrate greater potential in the field of synergistic therapy. In a breast cancer model, the multifunctional MOFs platform integrates chemotherapy, photothermal therapy, and immune activation modules, which not only improve the control of primary tumors but also stimulate systemic antitumor immune responses, thereby effectively inhibiting the formation of metastatic foci [34]. In colorectal cancer, MOFs constructed with copper‐olsalazine (Olsa) can regulate redox homeostasis, downregulate COX‐2 expression, and mediate epigenetic regulation, significantly inhibiting tumor growth and metastatic spread [14]. Furthermore, in the case of liver cancer, MOFs‐based nanoplatforms induce cuproptosis and ferroptosis, activate immune responses, and achieve dual effects in controlling both primary and metastatic foci [35]. In other types of tumors, such as lung cancer and gastric cancer, MOFs also demonstrate broad application potential in drug delivery, photothermal therapy, and immune modulation [36, 37].

## 3 Core Mechanisms of MOFs in Regulating Tumor Invasion and Metastasis

### 3.1 Tumor Metastatic Cascade: From EMT to Distant Colonization

**FIGURE 2:**
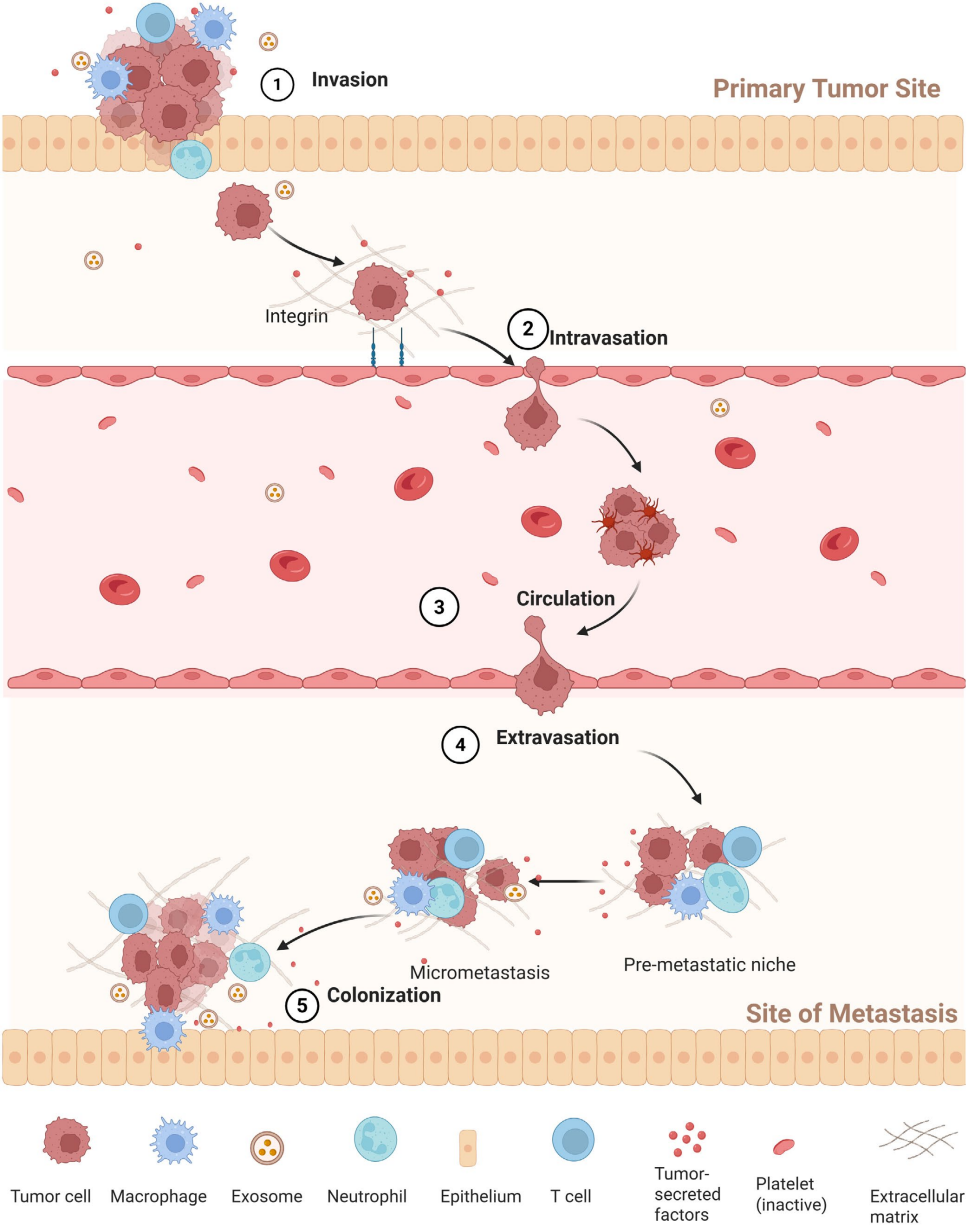
Overview of the metastatic cascade: The metastatic process involves five critical stages: initial invasion, entry into the bloodstream (intravasation), circulation through the body, exit from the vasculature (extravasation), and establishment of secondary tumors (colonization).

Tumor metastasis is a complex and highly coordinated multi‐step biological process, including EMT, local invasion, intravascular migration, survival in the bloodstream, adhesion and extravasation at distant organs, micrometastasis formation, and macroscopic colonization [38] (Figure 2). In the early stages of metastasis, EMT is considered a key step in initiating the migration and invasion abilities of tumor cells [7]. During this process, tumor epithelial cells lose cell polarity and intercellular junctions (such as the downregulation of E‐cadherin expression) and acquire mesenchymal‐like characteristics, including enhanced migratory and invasive abilities, as well as increased resistance to apoptosis [39]. Multiple signaling pathways, including TGF‐β, Wnt/β‐catenin, Notch, and Hippo pathways, can induce EMT‐related changes by activating transcription factors such as Snail, Slug, Twist, and the ZEB family [40].

Tumor cells that complete EMT subsequently traverse the basement membrane and ECM, entering the bloodstream or lymphatic circulation (i.e., intravascular metastasis) [41]. In the circulatory system, tumor cells must withstand fluid shear forces and immune clearance, often forming protective complexes by binding with platelets. These circulating tumor cells eventually become trapped in the capillary beds of distant organs, where they extravasate and adapt to the local microenvironment to complete colonization [42]. It is noteworthy that, before distant colonization, primary tumors can shape a pre‐metastatic niche (PMN) suitable for metastasis by secreting exosomes, cytokines, and bioactive molecules [43]. For example, lung cancer cells upregulate specific adhesion molecules, such as VCAM‐1 and integrins, promoting adhesion to pulmonary vascular endothelium, thereby preferentially forming metastatic foci in the lung tissue [44]. Overall, the success of tumor metastasis relies not only on the intrinsic plasticity of tumor cells but also on the co‐evolution of the microenvironment.

### 3.2 MOFs Interventions in Tumor Microenvironment, Cells, and Immune Targets

**FIGURE 3:**
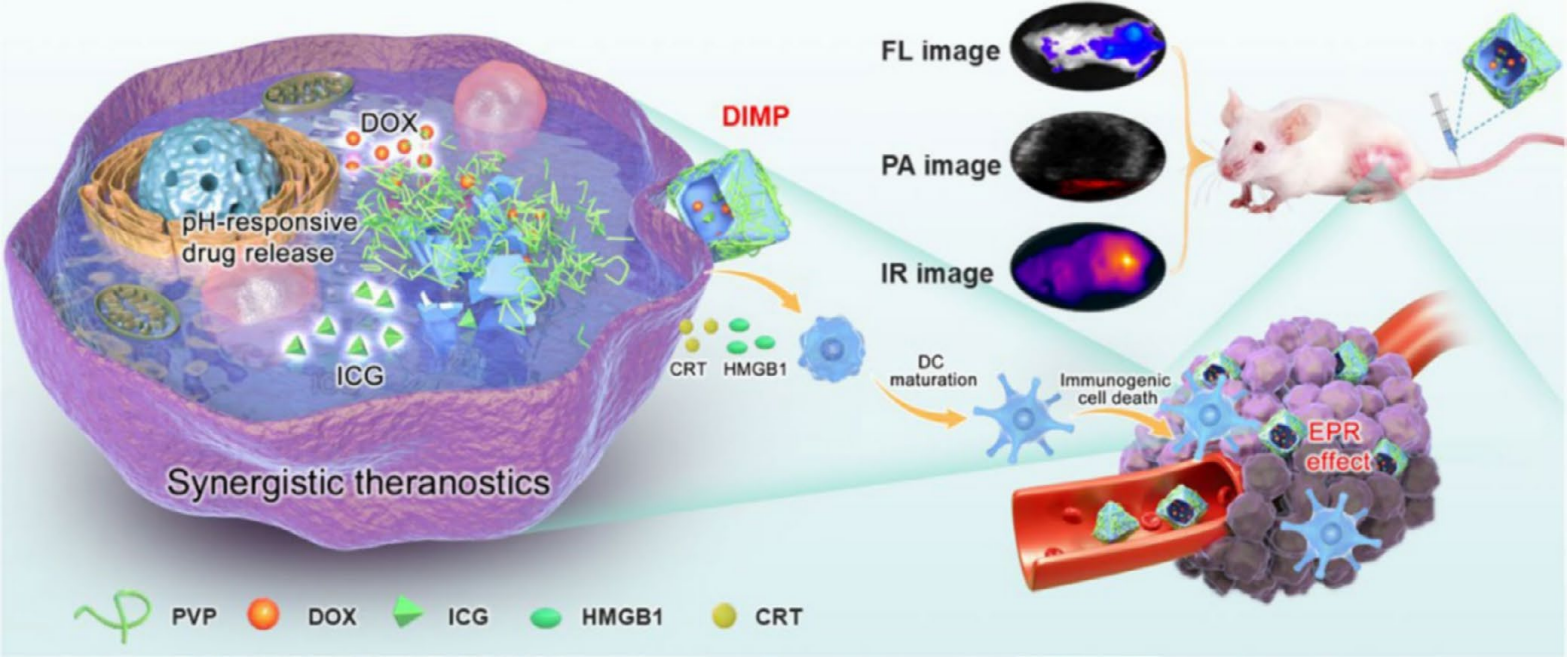
A polymeric metal–organic framework nanoparticle responsive to intracellular acidity, termed DIMP, was successfully constructed. Reprinted (adapted) with permission from [47]. Copyright © 2021, The Author(s). This scheme is licensed under a Creative Commons Attribution‐NonCommercial‐NoDerivatives 4.0 International License. To view a copy of this license, visit https://creativecommons.org/licenses/by‐nc‐nd/4.0/.

The tumor microenvironment (TME) plays a central role in regulating tumor invasion and metastasis. MOFs materials can intervene in the TME through multidimensional mechanisms, demonstrating immense potential in the prevention and treatment of tumor metastasis [45]. The TME exhibits characteristic changes, including acidity, hypoxia, high levels of reactive oxygen species (ROS), and immunosuppressive features, providing a unique responsive window for the design of MOFs [46]. For example, ZIF‐8‐type MOFs, due to their sensitivity to acidic environments, can undergo proton‐induced framework dissociation at tumor sites with a pH < 6.5, enabling localized drug release and thereby enhancing therapeutic efficacy while reducing systemic toxicity [47] (Figure 3).

In addition to drug delivery, MOFs can also remodel the tumor immune microenvironment. Certain MOFs structures, such as manganese‐based and redox‐active MOFs, can induce dendritic cell (DC) maturation, enhance antigen‐presenting ability, and activate CD8+ T cell‐mediated cytotoxic immune responses, effectively overcoming tumor immune evasion [48, 49]. Additionally, emerging evidence shows that MOFs can also regulate the polarization of tumor‐associated macrophages (TAMs), promoting the conversion of the immunosuppressive M2 phenotype to the pro‐inflammatory, antitumor M1 phenotype [50]. MOFs can also target stromal cells, such as CAFs, inhibiting their supportive role in tumor cells, thereby suppressing tumor invasion and metastasis [51, 52]. Therefore, by multidimensionally targeting the TME, MOFs provide a systematic intervention strategy for the prevention and treatment of tumor metastasis.

### 3.3 MOFs and Tumor Invasion and Metastasis‐Related Signaling Pathways

MOFs not only regulate the TME at the physicochemical level but also intervene in tumor cell invasion and metastasis by targeting key signaling or metabolic pathways, such as the mitochondria‐related cuproptosis mechanism [53]. Manganese‐based immunostimulatory MOFs (e.g., Mn@MOF) can activate the cytosolic DNA sensing pathway cGAS‐STING, induce type I interferon secretion, and activate immune cells such as dendritic cells and CD8+ T cells, thereby enhancing antitumor immune effects and inhibiting metastasis formation [50]. Copper‐olsalazine‐loaded nano MOFs target the downregulation of cyclooxygenase‐2 (COX‐2) and prostaglandin E2 (PGE2) signaling, inhibiting tumor cell proliferation and invasion, while reversing the immunosuppressive microenvironment, demonstrating significant antimetastatic potential [50].

Additionally, MOFs can serve as delivery platforms, carrying small interfering RNA (siRNA) or antisense oligonucleotides (ASO) to specifically inhibit EMT‐related transcription factors such as siSnail and siTwist. For example, using Zr‐based MOFs loaded with siSnail molecules allows for stable delivery and release at tumor sites, significantly inhibiting the EMT process and reducing tumor cell migration and metastasis ability at the source [54].

Through the aforementioned mechanisms, MOFs not only directly regulate key pathways within tumor cells but also block tumor metastasis occurrence and progression from multiple dimensions by immune remodeling and microenvironment intervention.

## 4 MOF‐Based Strategies for Antitumor Metastasis Therapy

Tumor metastasis is the leading cause of high cancer mortality, involving complex remodeling of the tumor microenvironment, cellular heterogeneity changes, and multi‐step signaling regulation [38]. MOFs, as emerging nanoplatforms, exhibit unique advantages in antimetastatic therapy due to their excellent structural tunability, diverse loading capabilities, and responsiveness [14]. This review systematically summarizes the research progress and innovative strategies of MOFs in microenvironment modulation, key cell intervention, multimodal combination therapy, and applications in specific tumor types.

### 4.1 MOF‐Based Tumor Microenvironment Modulation Strategies

TME such as hypoxia, acidity, immunosuppression, and extracellular matrix degradation, is a key factor promoting tumor metastasis. By selectively intervening in these abnormal features with MOF‐based materials, the pro‐metastatic effects of the TME can be effectively reversed.

Hypoxia, as a major inducer driving EMT and enhancing invasiveness, has become a key intervention target in tumor therapy. MOF‐based materials, with their ability to load catalytic active components such as MnO_2_, can promote H_2_O_2_ decomposition to generate oxygen, alleviating local tumor hypoxia and thereby improving therapeutic efficacy [55, 56, 57]. Additionally, acidic pH conditions not only promote tumor invasion but also act as a trigger for MOF‐based smart drug delivery. The design of pH‐sensitive MOF‐based systems, such as ZIF‐8 and MIL‐100, can rapidly degrade in the acidic tumor environment, precisely releasing chemotherapeutic drugs and improving local therapeutic efficacy [47]. On the other hand, ECM degradation also plays an important role in tumor cell migration. MOF‐based delivery systems can load MMP inhibitors to locally reduce the activity of MMP‐2 and MMP‐9, stabilize the matrix structure, and physically inhibit tumor cell invasion and dissemination [58, 59]. By integrating oxygen regulation, pH responsiveness, and matrix protection functions, MOFs offer a novel mode of multitarget synergistic intervention in microenvironment modulation.

### 4.2 MOF‐Based Strategies for Intervening and Clearing Key Metastatic Cells

**FIGURE 4:**
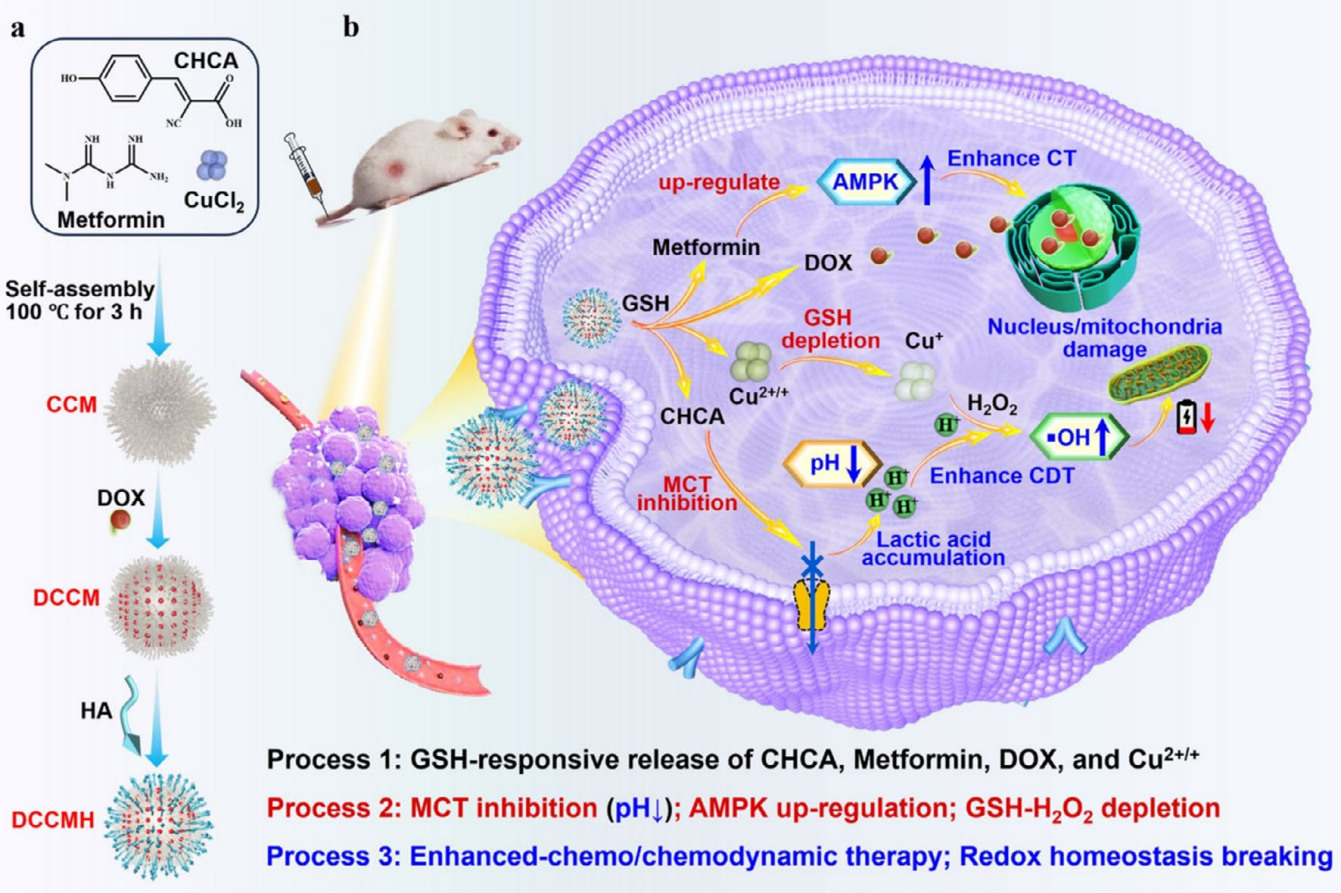
(a) Diagrammatic representation of the synthesis process for hyaluronic acid (HA)‐coated and doxorubicin (DOX)‐loaded coordination nMOFs. (b) Illustration of the mechanism underlying the dual chemo‐photothermal cancer therapy (DCCMH) for enhanced therapeutic efficacy. Reprinted (adapted) with permission from [62]. Copyright © 2025, The Author(s). License Information: This scheme is licensed under a Creative Commons Attribution‐NonCommercial‐NoDerivatives 4.0 International License. To view a copy of this license, visit http://creativecommons.org/licenses/by‐nc‐nd/4.0/.

The occurrence of tumor metastasis not only depends on microenvironment remodeling but is also closely associated with a series of key cell populations, such as circulating tumor cells (CTCs) and TAMs [60]. MOF‐based materials provide innovative approaches for the precise intervention and clearance of these cells [61] (Figure 4).

CTCs, as direct seeds of metastasis, require dynamic capture and elimination. By modifying the surface of MOFs with recognition molecules such as EpCAM and VCAM‐1, specific capture of CTCs can be achieved, and efficient clearance can be combined with photothermal therapy or immunotherapy [61]. Meanwhile, MOFs, as small nucleic acid delivery platforms (e.g., siRNA, miRNA), can precisely regulate EMT‐related transcription factors such as Snail and ZEB1, thereby reversing the migration ability of tumor cells [54].

Additionally, the polarization intervention of TAMs is gradually emerging as a new direction in antimetastatic strategies [63]. MOFs systems loaded with immunomodulators (such as CpG, TLR agonists) can promote the conversion of TAMs from the pro‐tumor M2 phenotype to the antitumor M1 phenotype, enhancing local tumor immune effects and further inhibiting the metastatic process [63, 64].

### 4.3 MOF‐Based Multimodal Combination Therapy Strategies

Given the inherent complexity of tumor metastasis, monotherapy is frequently inadequate in producing optimal therapeutic efficacy. The MOF‐based platform serves as an optimal integrative scaffold for multimodal synergistic therapies by orchestrating multiple therapeutic mechanisms to amplify overall treatment efficacy.

**FIGURE 5:**
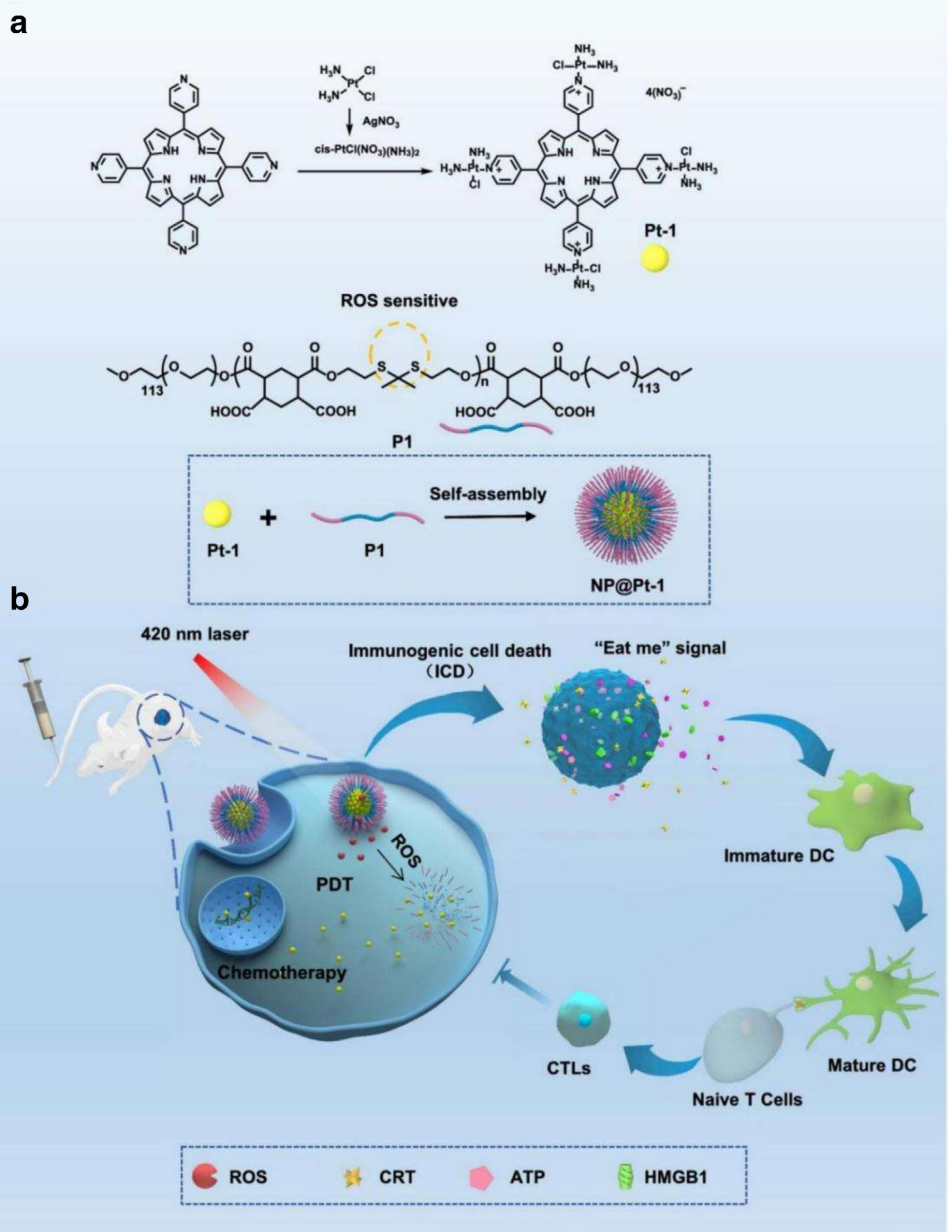
(A) Chemical structures and assembly of NP@Pt‐1. This panel depicts the chemical structures of Pt‐1 and the ROS‐sensitive polymer carrier (P1), along with their assembly into NP@Pt‐1 nanoparticles. (B) Mechanism of the triple therapeutic action of NP@Pt‐1. After administration to CT‐26 tumor‐bearing mice, NP@Pt‐1 accumulates at the tumor site. Upon light irradiation (420 nm), ROS generation induces cleavage of the polymer backbone (via thioketal bonds), resulting in the release of Pt‐1. Released Pt‐1 chelates intracellular DNA, triggering apoptosis and enabling chemotherapy. Additionally, ROS causes damage to cancer cell membranes, proteins, and DNA, contributing to PDT, while also inducing immunogenic cell death (ICD) by emitting “eat me” signals that promote cancer immunotherapy. Reprinted (adapted) with permission from [68]. Copyright © 2022, The Author(s). License Information: This figure is licensed under a Creative Commons Attribution‐NonCommercial‐NoDerivatives 4.0 International License. To view a copy of this license, visit http://creativecommons.org/licenses/by‐nc‐nd/4.0/.

MOFs offer a versatile platform for combinatorial cancer therapy. By simultaneously encapsulating angiogenesis inhibitors (e.g., Bevacizumab) and immunostimulants (e.g., CpG), they mediate dual regulation of the tumor microenvironment through vascular suppression and immune activation, impeding the development of pre‐metastatic niches [65]. Furthermore, as integrated delivery vehicles for photothermal, photodynamic, and chemotherapeutic agents, MOFs can generate hyperthermia, ROS, and controlled drug release upon NIR irradiation, achieving spatially targeted ablation of metastatic foci [66, 67] (Figure 5). These multimodal constructs exhibit markedly improved anti‐metastatic performance and hold promise for prolonging survival and reducing recurrence in preclinical models.

### 4.4 Targeted Utilization of MOFs in Representative Models of Tumor Metastasis

In response to the heterogeneous metastatic behaviors of various tumor types, a range of functionally tailored MOF‐based platforms has been engineered in recent years, significantly broadening their therapeutic and diagnostic potential.

In a lung cancer metastasis model, a dual MOF‐based system consisting of a PB@MIL88 core‐shell structure was engineered. This system facilitates the continuous release of hydroxyl radicals (·OH) through a self‐catalyzed Fenton reaction, leading to elevated ROS levels. When combined with chloroquine (CQ), which inhibits autophagy within the tumor cells, this strategy amplifies cytotoxicity and fosters T‐cell infiltration, ultimately achieving over 90% suppression of lung metastatic lesions [13, 67]. In a breast cancer bone metastasis model, Alendronate (Aln)‐modified Aln‐MOFs, by enhancing their affinity for bone tissue, facilitated the targeted delivery of doxorubicin (DOX) to the bone metastatic sites. In murine models, this system significantly delayed the progression of bone metastasis while increasing T‐cell recruitment efficiency, thus demonstrating significant synergistic effects in cancer treatment [69]. Additionally, for brain metastasis of small cell lung cancer (SCLC), a copper‐based Cu‐MOF nanodelivery system was engineered, incorporating stem cell membrane encapsulation and TP0751 peptide modification to facilitate efficient crossing of the blood–brain barrier (BBB). This system effectively delivered siRNA to silence ATP7a and induced cuproptosis. In vivo, it substantially inhibited the growth of brain metastatic lesions and significantly extended the survival of mice [70]. These studies underscore the exceptional tunability and targeted therapeutic potential of the MOF‐based platform, offering tailored and precise treatment strategies for a variety of metastatic cancers. The results demonstrate its substantial promise for future clinical applications and translation into therapeutic practice.

## 5 Therapeutic Approaches of MOFs in Tumor Invasion and Metastasis: Emerging Trends and Strategies

### 5.1 MOFs as Drug Delivery Systems

MOFs have emerged as highly promising platforms for drug delivery owing to their intrinsic high porosity and large specific surface area, which facilitate the efficient loading and controlled release of substantial quantities of therapeutic agents [71]. Numerous studies have demonstrated that MOFs can significantly enhance the drug loading capacity and improve the bioavailability of anticancer agents [72]. Moreover, the pore size and surface characteristics of MOFs are tunable, allowing them to respond to specific signals in the tumor microenvironment, such as pH and redox potential, thereby enabling the precise release of drugs [73]. This property endows MOFs with a distinct advantage in oncological applications, particularly in minimizing off‐target toxicity and reducing collateral damage to healthy tissues.

Through surface engineering, MOFs can also be endowed with active targeting capabilities [74]. For instance, functionalization with ligands such as folic acid enables MOFs to selectively recognize and bind to folate receptors, which are frequently overexpressed on the surface of various tumor cells. Such targeted delivery strategies enhance drug accumulation at the tumor site, improving therapeutic outcomes while minimizing systemic side effects [35]. Overall, MOF‐based drug delivery systems offer an innovative and versatile approach for improving both the efficacy and safety of cancer therapies [72].

### 5.2 The Application of MOFs in Photothermal and Photodynamic Therapy

**FIGURE 6:**
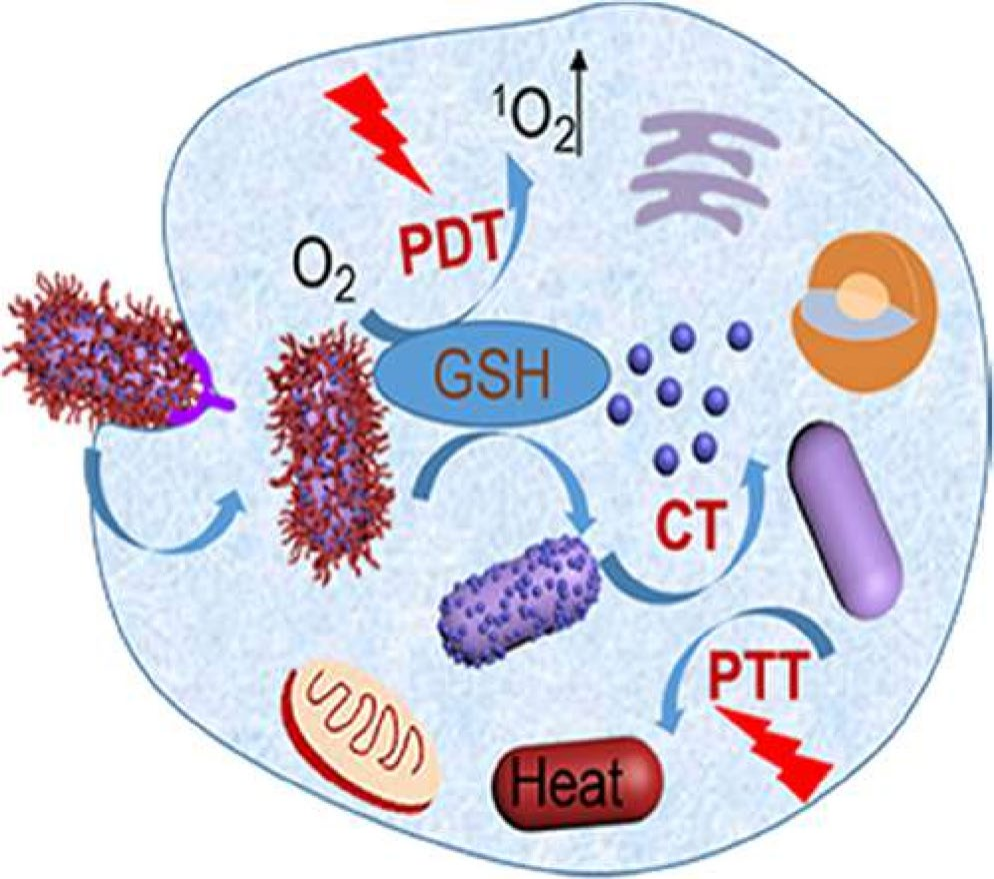
Combination therapy integrating PTT and PDT to achieve synergistic antitumor effects. Reprinted (adapted) with permission from [76]. Copyright © 2022, American Chemical Society.

PTT and PDT have attracted substantial interest as emerging strategies for tumor treatment [75]. Certain MOFs exhibit exceptional photothermal conversion capabilities, enabling the transformation of near‐infrared (NIR) light into localized heat, thereby inducing tumor cell ablation [76] (Figure 6). Studies have demonstrated that MOF materials composed of specific metal ions or organic ligands can achieve high photothermal conversion efficiency, making them highly effective for thermal destruction of tumor tissues [72].

In the context of PDT, MOFs serve not only as nanocarriers for photosensitizers but also as intrinsic photosensitizing agents themselves [77]. Upon exposure to light of appropriate wavelengths, these MOFs generate singlet oxygen and other ROS, which in turn induce apoptosis in tumor cells. The integration of PTT and PDT can result in a synergistic therapeutic effect, significantly enhancing overall treatment efficacy [78, 79] For instance, certain MOFs irradiated with NIR light can concurrently ablate tumor cells via hyperthermia and generate cytotoxic ROS via photodynamic processes, thereby markedly improving antitumor outcomes [80, 81]. This multifunctional and synergistic therapeutic platform represents a promising strategy for precision oncology.

### 5.3 The Combination of MOFs and Immunotherapy

The integration of MOFs with immunotherapy has unveiled promising new avenues for combating tumor invasion and metastasis [82]. MOFs not only function as delivery vehicles for immune adjuvants or modulators, enabling the precise transport of immunostimulatory agents, but also contribute to the amplification of immune responses within the tumor microenvironment [81]. For instance, studies have demonstrated that loading immune stimulators such as CpG oligodeoxynucleotides onto MOFs significantly enhances antitumor immunity [83]. The intrinsic porous architecture of MOFs protects these stimulators from premature degradation and facilitates their controlled release at the tumor site, thereby promoting the activation of immune cells and boosting their cytotoxic activity against tumor cells [46].

**FIGURE 7:**
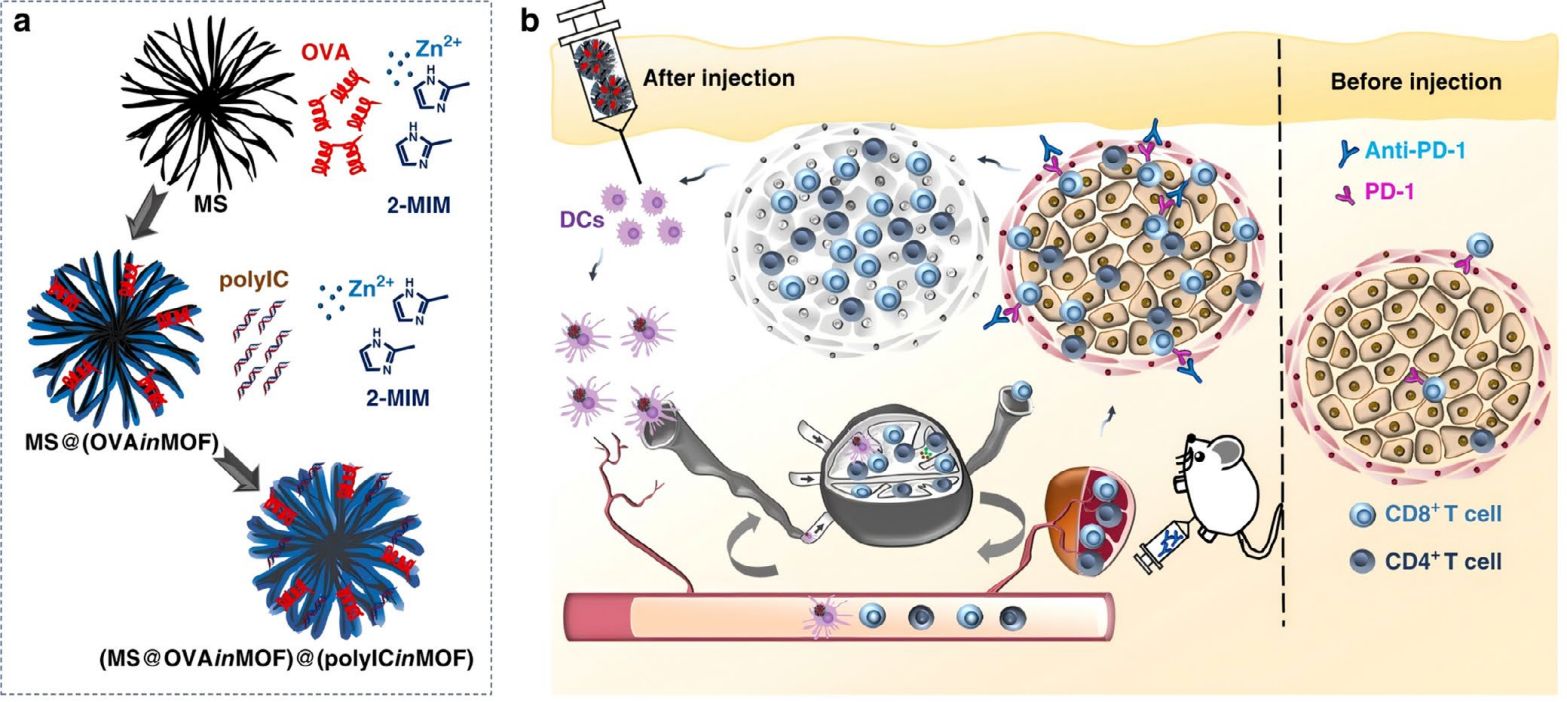
(a) Schematic representation of the fabrication process of (MS@OVAinMOF)@(polyICinMOF). (b) Illustration of the mechanism of the MOF‐gated MS cancer vaccine in combination with anti‐PD‐1 Ab. This combination stimulates the activation of DCs, enhances cancer antigen‐specific T‐cell activity in the lymph nodes and spleen, releases immunological brakes, and inhibits tumor growth. Reprinted (adapted, with cropping of Figure 5 to include only parts a and b for clarity) with permission from [85]. Copyright © 2020, The Author(s). This scheme is licensed under a Creative Commons Attribution‐NonCommercial‐NoDerivatives 4.0 International License. To view a copy of this license, visit http://creativecommons.org/licenses/by‐nc‐nd/4.0/.

Furthermore, MOF‐mediated photothermal and photodynamic therapies can induce immunogenic cell death, leading to the release of tumor‐associated antigens and activation of the adaptive immune response. This enhances immune surveillance and facilitates the targeted elimination of malignant cells [84]. By incorporating immune checkpoint inhibitors and other immunotherapeutic modalities, MOFs can effectively disrupt tumor immune evasion mechanisms, further augmenting the efficacy of immunotherapy [85, 86] (Figure 7). Accumulating evidence indicates that the synergy between MOFs and immunotherapeutic strategies yields significant therapeutic benefits across multiple tumor models, highlighting their potential to reshape the landscape of cancer immunotherapy and robustly enhance antitumor immune activation [87, 88].

## 6 Controversies and Future Prospects of MOFs in Tumor Invasion and Metastasis

### 6.1 Biosafety Issues of MOFs

Although MOFs have shown considerable promise in the treatment of tumor invasion and metastasis, concerns regarding their biosafety remain a critical barrier to clinical application [89]. The primary safety concerns relate to the potential toxicity of their constituent metal ions and organic ligands, the biological effects of their degradation products, and their immunogenic potential [72, 90]. The toxicity of metal ions varies depending on their type and concentration; for instance, ions such as cobalt and nickel may exert deleterious effects at elevated levels. Likewise, the biocompatibility of organic ligands remains insufficiently understood, particularly with regard to their metabolic pathways and in vivo toxicity profiles [91]. Furthermore, MOFs' degradation products may interact with cellular and tissue components, raising additional safety concerns that require thorough evaluation [92]. As exogenous materials, MOFs may also elicit immune responses. Although some studies have indicated favorable biocompatibility for certain MOFs, comprehensive investigations are still needed to assess their long‐term immunogenicity [93]. Resolving these biosafety challenges is an essential prerequisite for the successful clinical translation of MOF‐based therapies.

### 6.2 Challenges of MOFs in Clinical Translation

Despite significant progress in laboratory research, the clinical translation of MOFs still faces considerable challenges. One of the foremost obstacles lies in the large‐scale synthesis of high‐quality, homogeneous MOF‐based materials. Existing synthetic approaches often fall short of meeting clinical demands in terms of both quantity and consistency, with issues related to reproducibility and material stability remaining unresolved [94, 95]. Moreover, the pharmacokinetic and pharmacodynamic profiles of MOFs in vivo are not yet fully characterized. Comprehensive studies on their absorption, distribution, metabolism, and excretion are essential to define optimal administration routes and dosing regimens [96]. In parallel, the molecular mechanisms underlying MOFs' interactions with biological systems warrant further elucidation, including their binding affinity for cell surface receptors, modes of cellular uptake, and influence on intracellular signaling pathways [97]. Regulatory considerations also pose a significant hurdle, underscoring the urgent need for standardized evaluation frameworks and approval procedures to ensure the safety and therapeutic efficacy of MOF‐based interventions [98].

### 6.3 Future Directions of MOFs in Tumor Invasion and Metastasis Research

In the future, MOFs are expected to achieve significant advances in the study of tumor invasion and metastasis. To this end, continued refinement in the design and synthesis of MOFs is essential for the development of novel materials exhibiting superior biocompatibility, enhanced targeting capabilities, and improved therapeutic efficacy [99]. For instance, precise control over MOFs' structure and composition enables the synergistic integration of multiple therapeutic modalities, thereby augmenting the precision and effectiveness of cancer treatments [100]. Moreover, elucidating the mechanisms underlying the interactions among MOFs, tumor cells, and the tumor microenvironment will provide a theoretical foundation for more targeted therapeutic strategies [101]. The incorporation of advanced imaging technologies can further facilitate real‐time monitoring and feedback of MOF‐based therapeutic processes, thus optimizing treatment outcomes [102]. Furthermore, integrating MOFs with cutting‐edge therapeutic platforms such as gene editing and cell therapy holds great promise for expanding therapeutic options and driving novel breakthroughs in combating tumor invasion and metastasis [103].

### 6.4 MOFs Vs. Lipid and Polymeric Nanocarriers in Anti‐Metastatic Therapy

In anti‐metastatic therapy, a comparative analysis between MOFs and conventional nanocarriers such as lipid nanoparticles and polymeric nanoparticles reveals significant differences in their properties and potential applications [104, 105].

While lipid nanoparticles, such as solid lipid nanoparticles and nanostructured lipid carriers, are widely used for drug delivery due to their excellent biocompatibility and targeting ability [106, 107], they often face challenges like limited drug‐loading capacity and instability, particularly when delivering high doses or multiple drugs simultaneously. In contrast, MOFs offer higher drug‐loading efficiency and the ability to respond to multiple stimuli, providing a solution to these limitations [104, 105].

Polymeric nanoparticles, known for their excellent mechanical strength and stability, are commonly used in drug delivery. Recent advances have led to hybrid systems that combine lipid and polymeric nanoparticles to enhance stability and targeting [108]. However, in complex tumor microenvironments, MOFs demonstrate superior adaptability due to their modular structure and tunability. MOFs can also be integrated with polymeric materials to form hybrid nanoplatforms, improving stability and adding therapeutic functionalities [109].

Therefore, while conventional lipid and polymer nanoparticles have established clinical applications and offer stability, MOFs possess unique advantages that can complement or even replace these carriers. With proper design, MOFs may serve as a superior alternative or synergize with conventional nanocarriers to enhance therapeutic efficacy.

### 6.5 MOFs‐Mediated Metastasis Inhibition Strategies: From Multi‐Target Intervention to Clinical Translation

MOFs mediated metastasis inhibition strategies are advancing toward multi‐target intervention and clinical translation [110]. In terms of multi‐target intervention, multifunctional MOFs materials are engineered to simultaneously address multiple critical stages in the tumor metastasis process. These include modulating tumor cell migration, targeting invasion‐related signaling pathways, and influencing immune and stromal cells within the tumor microenvironment, thereby achieving a more comprehensive and effective inhibition of metastasis [33, 111].

Regarding clinical translation, the biosafety concerns and clinical challenges previously discussed must be resolved. Further preclinical studies are essential to rigorously evaluate the safety and efficacy of MOFs, as well as to optimize treatment protocols [112]. In parallel, strengthening collaborations with clinicians, conducting clinical trials, and advancing MOFs‐mediated metastasis inhibition strategies from laboratory research to clinical practice will offer more effective treatment options for cancer patients and improve patient outcomes.

## 7 Conclusion

MOFs, as a novel class of nanomaterials, have demonstrated tremendous potential in the treatment of tumor invasion and metastasis. Owing to their high porosity, tunable pore size, and modifiable surface chemistry, MOFs can efficiently load and deliver anticancer agents, thereby enhancing both treatment efficacy and specificity. Their versatility also supports multimodal therapies, including PTT, PDT, and immunotherapy, offering synergistic antitumor strategies.

Despite these advantages, the clinical translation of MOFs remains challenging. Key barriers include unresolved biosafety concerns, such as the potential toxicity of metal ions and the immunogenicity of their degradation products. In addition, the limited understanding of their pharmacokinetics and interactions within biological systems presents further obstacles. The complexity of large‐scale synthesis may also hinder reproducibility and limit clinical scalability.

Looking forward, optimizing the physicochemical design of MOFs to enhance biocompatibility, tumor targeting, and in vivo stability is crucial. Integration with emerging therapeutic modalities such as gene editing, cell therapy, and multi‐target delivery could further strengthen their therapeutic potential. In addition, the application of real‐time imaging and responsive monitoring technologies may enable personalized, adaptive treatments based on individual tumor profiles.

In summary, MOFs represent a highly adaptable and multifunctional platform for combating tumor invasion and metastasis. With continued innovation and interdisciplinary collaboration, they are poised to offer more effective and personalized treatment options, ultimately improving clinical outcomes and patient quality of life.

## Author Contributions

Jiahui Wang: writing – original draft, writing – review and editing, visualization, investigation. Xiuxiu Qiu: writing – original draft, writing – review and editing. Ruoyu Wang: investigation, visualization. Beibei Hong:conceptualization, supervision. Hegen Li: writing – review and editing, supervision, funding acquisition. Zhanxia Zhang: writing – review and editing, visualization, investigation, funding acquisition, project administration.

## Ethics Statement

The authors have nothing to report.

## Conflicts of Interest

The authors declare no conflicts of interest.

## Data Availability

This review does not involve the generation of new experimental data. The information presented is derived from published studies and reports, and no original data were collected or analyzed for this review. Therefore, a data statement is not applicable.
